# Identification and picking point positioning of tender tea shoots based on MR3P-TS model

**DOI:** 10.3389/fpls.2022.962391

**Published:** 2022-08-12

**Authors:** Lijie Yan, Kaihua Wu, Jia Lin, Xingang Xu, Jingcheng Zhang, Xiaohu Zhao, James Tayor, Dongmei Chen

**Affiliations:** ^1^School of Automation, Hangzhou Dianzi University, Hangzhou, China; ^2^Beijing Research Center for Information Technology in Agriculture, Beijing Academy of Agriculture and Forestry Sciences, Beijing, China; ^3^School of Natural and Environmental Sciences, Newcastle University, Newcastle Upon Tyne, United Kingdom

**Keywords:** image analysis, deep learning, *Camellia sinensis*, faster region-based convolutional neural network, fine tea picking

## Abstract

Tea is one of the most common beverages in the world. In order to reduce the cost of artificial tea picking and improve the competitiveness of tea production, this paper proposes a new model, termed the Mask R-CNN Positioning of Picking Point for Tea Shoots (MR3P-TS) model, for the identification of the contour of each tea shoot and the location of picking points. In this study, a dataset of tender tea shoot images taken in a real, complex scene was constructed. Subsequently, an improved Mask R-CNN model (the MR3P-TS model) was built that extended the mask branch in the network design. By calculating the area of multiple connected domains of the mask, the main part of the shoot was identified. Then, the minimum circumscribed rectangle of the main part is calculated to determine the tea shoot axis, and to finally obtain the position coordinates of the picking point. The MR3P-TS model proposed in this paper achieved an mAP of 0.449 and an *F*2 value of 0.313 in shoot identification, and achieved a precision of 0.949 and a recall of 0.910 in the localization of the picking points. Compared with the mainstream object detection algorithms YOLOv3 and Faster R-CNN, the MR3P-TS algorithm had a good recognition effect on the overlapping shoots in an unstructured environment, which was stronger in both versatility and robustness. The proposed method can accurately detect and segment tea bud regions in real complex scenes at the pixel level, and provide precise location coordinates of suggested picking points, which should support the further development of automated tea picking machines.

## Introduction

The tea plant (*Camellia sinensis*) is grown to produce tea, a popular beverage, worldwide. There are many processing steps in tea production, which vary with the different types of tea produced, but tea harvesting is an essential prerequisite for all types of production (Tian et al., 2021). Traditionally, tea harvesting has been performed by hand. However, with the increasing labor cost, lack of specialists, and higher quality requirements from tea producers, mechanized tea picking is becoming an inevitable trend for the sustainable development of the tea industry (Zhu et al., 2021).

Mechanized tea harvesters have been researched and developed in many countries to conform to local production conditions, but harvesters can be mainly classified into a reciprocating cutting type, spiral hob type, horizontal-circle blade type, or spiral roll folding type (Han et al., 2014, 2019). Harvesters can reduce the cost and improve the efficiency of the tea harvest; however, they usually cut the top leaves without distinguishing between the desirable young shoots and older leaves. This leads to the harvested crop containing old and broken leaves and a lower quality of product (Madamombe et al., 2015). At the same time, mechanical harvesters may also damage the stems of trees and affect the germination of new shoots, and potential yield, for the next harvest (Abesinghe et al., 2020).

To improve crop quality from mechanized picking, the first and essential task is to be able to recognize the tender tea shoots and accurately localize the picking points in a complex vegetative system (Li et al., 2021). The more tender the leaves are, the higher the quality and the price achieved. The optimal picking situation is considered to be a single tip with two leaves (Madamombe et al., 2015; Yang et al., 2021). Some traditional image processing-based methods have been proposed to identify these two-leaf tips to achieve such a task (Chen et al., 2015; Ke and Lv, 2016). Wu et al. (2015) detected new tea bud leaves (tips) from among older leaves using color transformation, Otsu’s thresholding, and *k*-means clustering. Thangavel and Murthi (2017) counted the number of tea shoots using key frame extraction, rice counting, optical flow, and the Prewitt operator. Karunasena and Priyankara (2020) applied a machine learning object detection technique to identify tea bud leaves and achieved 55% of overall accuracy, while Shao et al. (2018) segmented young leaves in tea images with the HSI color model and the improved *k*-means algorithm. However, the above research actions were performed under controlled conditions and poorly replicated the real conditions within tea fields with complex environments, including uncontrolled illumination and a high level of similarity between the foreground and background (Chen and Chen, 2020).

In comparison to the methods above, convolutional neural networks (CNN) constitute a deeper neural network that provides a hierarchical representation of the data with various convolutions (Lecun and Bengio, 1995; Schmidhuber, 2015). CNN models have shown remarkable performance in various imagery-related problems in agriculture with complex background, including target recognition and detection in unstructured environments (Kamilaris and Prenafeta-Boldú, 2018; Yu et al., 2019). Yang et al. (2019) trained a tea shoot detection model with the improved “you only look once” (YOLO) network and achieved a high accuracy for the validation data set. Chen et al. (2018) achieved tea shoot detection using a faster region-based convolutional neural network (Faster R-CNN), which Chen and Chen (2020) coupled with a fully convolutional network (FCN) to identify the picking point on the tea shoot region. However, the tea shoot picking point detection model of Chen and Chen (2020) is a two-stage model with complicated detection and segmentation steps. Another tea shoot segmentation model, based on the improved deep convolutional encoder-decoder Network (TS-SegNet) with a contrastive-center loss function and skip connections, has also been proposed (Qian et al., 2020), but this model can only realize the semantic segmentation of the tea without distinguishing different tea shoots.

Therefore, there is still an absence of a model that can directly detect the contour of each tea shoot in the image and produce the accurate location of the tea shoots’ picking points. In this paper, a Mask R-CNN Positioning of Picking Point for Tea Shoots (MR3P-TS) model is proposed for the identification and picking point positioning of tender tea shoot. The main contributions are as follows: (1) separation of different tea shoots under the complex field background; (2) accurate extraction and counting of the edge information of tea shoots; and (3) end-to-end output of the picking point position coordinates and suggested knife angle.

## Materials and methods

### Image acquisition

**Figure 1:**
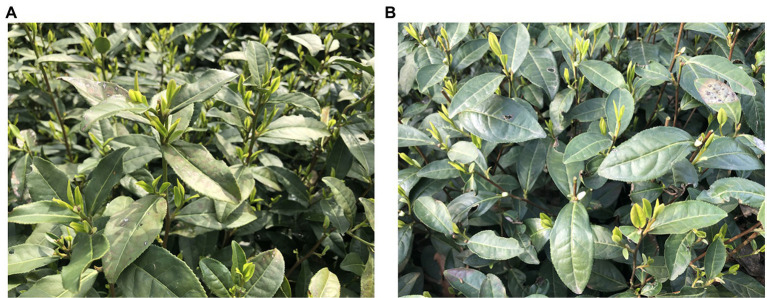
Examples of the 1,058 images collected in the study in a natural environment under **(A)** sun-lit conditions and **(B)** cloud-covered conditions.

The experimental images were collected in several tea gardens in the West Lake scenic area, Hangzhou, Zhejiang province, China. The target variety was “Longjing 43,” which was bred by the tea research institute of the China agricultural science park. In the study, we took RGB images of tea buds with the iPhone rear camera at a distance of 60–80 cm from the tea tree with multiple angles in March 2021. The dual rear cameras we used are divided into wide-angle and ultra-wide-angle lenses. The wide-angle is a 1,200 W pixel camera with F1.6 + 7P lens OIS + dual-core focus, while the ultra wide-angle is a 1,200 W pixel lens with an equivalent focal length of 13 mm + 120° + F2.4. In order to ensure that the model had a good generalization ability and robustness, the image acquisition process included two different light scenarios: under sunny and cloudy conditions. The storage format was JPG. The original images were cropped in a 2*4 ratio to limit the number of shoots per image. After cropping, the pixel resolution of the tea shoot image used in this paper was 1512*1008. The images were visually assessed, and those with a clear shoot outline and a visible picking point were retained, resulting in a final dataset of 464 images. Examples of the acquired images are shown in Figure 1.

### Data set construction and annotation

In order to prevent the model from overfitting during the training process, image augmentation was used to expand the dataset. The image-augmented data reduced the unbalanced distribution of samples and improved the generalization ability of the model. The methods of image augmentation used in the study mainly included scale transformation, flip transformation, and pixel value normalization.

**Figure 2:**
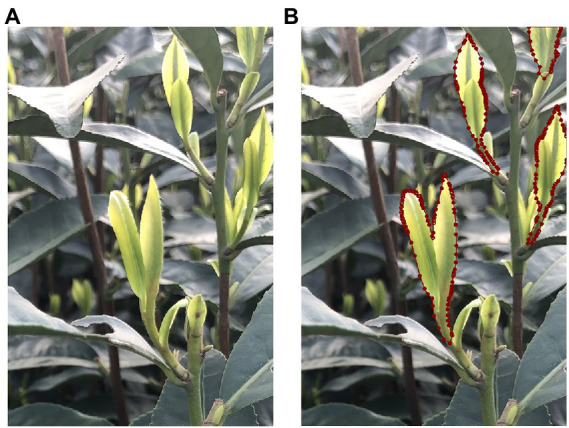
Instance segmentation example of tea shoots dataset **(A)** original image, **(B)** the visualization of the boundary points for the mask.

After data augmentation processing, the imagery dataset was randomly divided into three groups, with the ratio of 70, 20, and 10%, to form the model training, model validation, and testing datasets, respectively. The training set was used to learn the weight parameters in the model training process, the validation set was used to optimize the network model structure, and the testing set was used to verify the accuracy of the proposed method. The image data of tea shoots were annotated by LabelMe (Russell, 2008), an open-source annotation tool from the Massachusetts Institute of Technology (MIT), to generate masks of the tea shoots. These masks were used for model training and parameter optimization, and the inverse loss was calculated and compared with the predicted results to evaluate the instance segmentation performance of the model. The tea shoot area in the image was labeled, and the rest of the image was denoted as the background by default. An example of a raw and annotated tea shoot image is shown in Figure 2.

### MR3P-TS model

**Figure 3:**
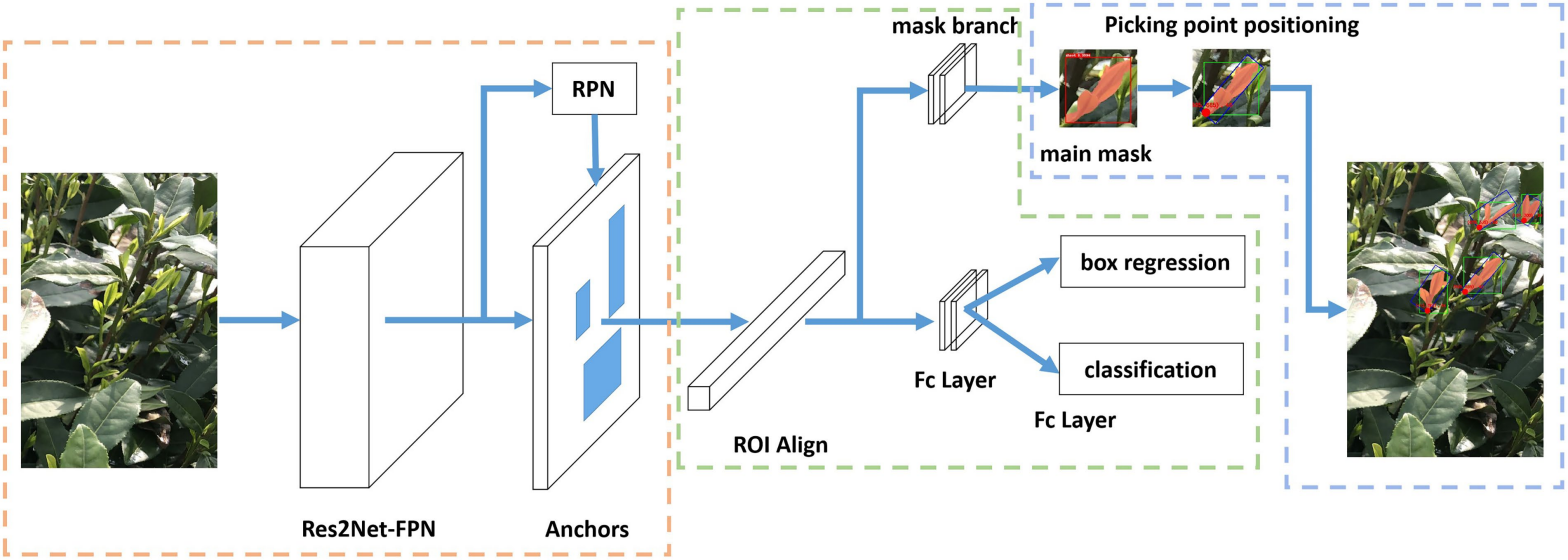
Schematic overview of the complete Mask R-CNN Positioning of Picking Point for TeaShoots (MR3P-TS) model structure.

An overview of the proposed method, the MR3P-TS model for tea shoot identification and picking point positioning, is shown in Figure 3. The MR3P-TS model was extended from the Mask R-CNN framework (He et al., 2017) and can be divided into three stages. In the first stage, the backbone network is used to extract feature maps from the input image, and then, the feature maps are sent to the region proposal network (RPN) to generate regions of interest (RoIs). In the second stage, RoIs are mapped to the feature map to extract the corresponding target features, which are sent to the head network to predict the target box and mask. The head network includes a fully connected layer (FC layer) and a fully convolutional network (FCN). The third stage is the positioning method of the picking point. The mask obtained in the second stage is subjected to maximum connected domain processing to obtain the main part of the shoot. Then, the minimum circumscribed rectangle of the main body is calculated to determine the axis of the shoots from which the position coordinates of the picking point can be determined.

#### Feature extraction and generation of regions of interest

The backbone network is usually a neural network with a certain depth to extract feature maps from the input image. When the number of network layers is deepened, the model’s expressive ability does not always theoretically strengthen, and the model will degenerate, that is, the network will converge slowly and the training accuracy will decrease (He and Jian, 2015; Srivastava et al., 2015). The emergence of the residual network model (ResNet; He et al., 2016) has solved this problem. As a network structure with cross-layer connection, ResNet builds a residual structure on the basis of the VGG model, and connects the shallow network and the deep network across layers to enable deep training. Errors can be back-propagated to shallow layers, effectively alleviating the issue of training degradation. As an improvement of ResNet, Res2Net (Gao et al., 2019) adds a small residual block to the original residual unit structure, which enables the network to represent multi-scale features at a finer granularity and increases the receptive field of each layer of the network.

Feature pyramid network (FPN), as a feature extraction network, can be used to assist ResNet in feature extraction. Using a top-down architecture with horizontal connections, FPN can fuse feature maps with strong low-resolution semantic information and feature maps with weak high-resolution semantic information by providing rich spatial information with less computation. This makes feature maps of different sizes in each layer of the FPN network that have strong semantic information, and allows the prediction of the feature maps of each layer separately.

The feature map output by the backbone network is used as the input of the Region Proposal Network (RPN) to extract candidate boxes, and then, the boxes generate Regions of Interest (RoIs). The RPN network is a module specially used to extract candidate boxes. It slides over the feature map through nine anchor boxes of different scales and proportions and further filters the anchor boxes according to the foreground score before using the bounding box regression parameters to correct the anchor boxes. Anchor boxes that cross the image boundary after correction are discarded. During the training process, each image generated too many RoIs. In the study, RoIs with an Intersection over Union (IoU) greater than 0.7 by non-maximum suppression were removed. The threshold of 0.7 was chosen based on expert experience with these images.

#### Target detection and instance segmentation

In order to map the RoIs to the feature map to extract the corresponding target features, a region of interest alignment (RoI Align) method was used to replace the region of interest pooling (RoI Pooling) in the Faster R-CNN network. Pixel-level segmentation was achieved by using a mask branch, on the premise that the precise position of the input feature can be obtained. RoI Align eliminates the quantization operation and does not quantify the RoI boundary and unit. It preserves the decimal point and then uses bilinear interpolation to calculate the exact location of the sampling point in each unit, and uses max pooling or average pooling to output the final fixed-size RoI. Finally, it uses the head network to make predictions, including an FC layer for classification prediction, a regression layer for bounding box coordinate correction, and an FCN for instance segmentation to generate object masks.

#### Position of the tea shoots picking point

**Figure 4:**
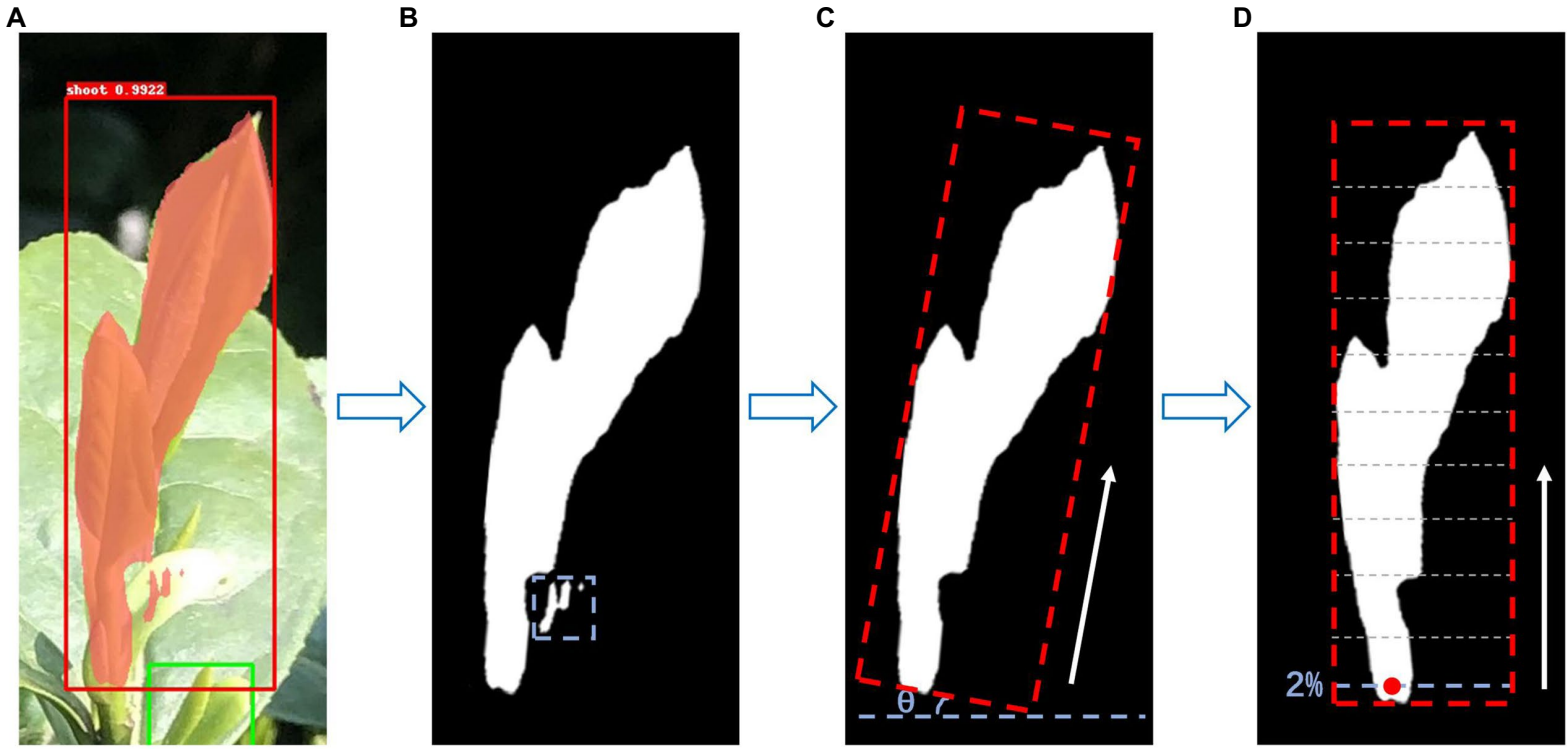
Implementation of picking point determination **(A)** the result of the contour of shoot identification, **(B)** the determination of the shoot main mask, **(C)** calculation of the minimum circumscribed rectangle, and **(D)** calculation of the 2D coordinate information of the picking point.

While maintaining the classification and bounding box regression, a parallel branch is added in the model structure (Figure 3) to output a binary mask that reflects the position and shape of the target object in the RoIs. Tea shoots generally grow vertically, and the picking points are generally distributed below the intersection of the shoot axis and the leaf. Therefore, the basis for positioning the tea picking point is to determine the direction of the shoot axis. Through empirical observation, it was found that the picking points are often located at a point of approximately 2% of the total shoot length starting from the bottom of the masked area. The final output also includes a suggested knife angle, i.e., the inclination angle of the shoot axis to the knife, which can meet the requirements of a simple shoot picking robot. The whole process of selecting the picking point is shown in Figure 4. Firstly, by looking for the maximum connected domain in the shoot identification result (Figure 4A), the picking point prediction regions are located, which are called as the main mask (Figure 4B), because picking points are often more likely to be found in connected domains with larger areas. The minimum bounding rectangle of the main mask is calculated to obtain the shoot axis and the suggested knife angle (Figure 4C). The shoot axis is taken from the rotation angle of the minimum bounding rectangle, and the angle θ between the bottom edge of the rectangular box and the shoot axis is the suggested knife angle. Finally, the position of the picking point, at 2% of the total mask length starting from the bottom, is identified (Figure 4D).

#### Loss function

The loss function of the model consists of two parts, namely the RPN network loss and the head network loss. The loss function is defined as follows:


$$L=L_{RPN}+L_{head}.$$

Among them, *L*_RPN_ contains classification loss and bounding box regression loss, which is:


$$\begin{array}{c} L_{RPN}=L_{cls}+L_{reg}=\frac{1}{N_{cls1}}\underset{i}{\sum }L_{cls}\left(p_{i},p_{i}^{*}\right)\\ +\lambda_{1}\frac{1}{N_{reg1}}\underset{i}{\sum }p_{i}^{*}L_{reg}\left(t_{i},t_{i}^{*}\right). \end{array}$$

In the formula, *i* represents the anchor index, *p_i_* represents the positive softmax probability, p_i_^*^ represents the corresponding GT predict probability. When IoU > 0.7 between the *i*th anchor and GT, the anchor is supposed to be positive, p_i_^*^ = 1. When IoU < 0.3, the anchor is supposed to be negative, p_i_^*^ = 0. As for those anchors with 0.3 < IoU < 0.7, they do not participate in training. *t* represents the predict bounding box, t^*^ represents the GT box corresponding to the positive anchor, and λ_1_is an adjustable parameter used to balance the number of anchors *N*_cls1_and the number of bounding boxes *N*_reg1_. *L*_cls_ uses the binary cross-entropy loss, and *L*_reg_ uses the smooth_L1_ loss.

*L*_head_ contains classification, bounding box regression and mask loss, which is:


$$\begin{array}{c} L_{\mathrm{head}}=\frac{1}{N_{\mathrm{cls}2}}\underset{i}{\sum }L_{\mathrm{cls}}\left(p_{i},p_{i}^{*}\right)+\lambda_{2}\frac{1}{N_{\mathrm{reg}2}}\underset{i}{\sum }p_{i}^{*}L_{\mathrm{reg}}\left(t_{i},t_{i}^{*}\right)\\ +\gamma \frac{1}{N_{\mathrm{mask}}}\underset{i}{\sum }L_{\mathrm{mask}}\left(s_{i},s_{i}^{*}\right). \end{array}$$

In the formula, *s* represents the binary predict mask, s^*^ represents the GT mask of the corresponding category. *L*_mask_ is the binary cross-entropy loss of a single category, and only calculates the loss of the corresponding category for each pixel, avoiding competition between classes. The calculation formulas of classification loss *L*_cls_ bounding box regression loss *L*_reg_ and mask loss *L*_mask_ are as follows:


$$L_{\mathrm{cls}}\left(p_{i}^{*},p_{i}\right)=-p^{*}\log p$$

$$\begin{array}{c} L_{\mathrm{reg}}\left(t_{i}^{*},t_{i}\right)={\mathrm{smooth}}_{L1}\left(t^{*}-t\right),\\ {\mathrm{smooth}}_{L1}\left(x\right)=\{\begin{array}{c} 0.5x^{2}\;\mathrm{if}\left|x\right|<1\\ \left|x\right|-0.5\;\mathrm{otherwise} \end{array}. \end{array}$$

$$L_{\mathrm{mask}}\left(s_{i}^{*},s_{i}\right)=-\left(S^{*}\mathrm{logs}+\left(1-s^{*}\right)\log\left(1-s\right)\right).$$

### Evaluation

#### Evaluation of tea shoots identification

For the bounding box regression and mask output by the model, F_β_ was chosen as the performance metric to evaluate the shoot identification problem. F_β_ can express the different preferences of the task for precision mAP and recall mAR and is defined as:


$$F_{\beta }=\frac{\left(1+\beta \right)\times mAP\times mAR}{\left(\beta^{2}\times mAP\right)+mAR}.$$

In the task of machine picking, the picking machine needs to capture images from multiple angles of the same cluster of tea trees, in order to alleviate the classic problem that the shoots are occluded and cannot be correctly identified due to the dense growth of tea leaves. The increase of the acquisition angle makes it more important to pay attention to whether the shoots can be correctly identified when evaluating the model, rather than missing the shoots as little as possible. So, the contribution of the precision rate to the performance measurement should be greater. Based on experience and the actual meaning of each parameter, we finally decided to set *β* to 2.

The IoU evaluates the overlap between the generated candidate box (Candidate bound, C) and the ground truth bound (ground truth bound, G), which is defined as follows:


$$\mathrm{IoU}=\frac{area\left(c\right)\cap area\left(G\right)}{area\left(c\right)\cup area\left(G\right)}.$$

mAP and mAR are an average concept, which is the average of each precision rate and recall rate when IoU takes [0.45:0.05:0.95], which is defined as follows:


$$\mathrm{mAP}=\frac{1}{11}\overset{0.95}{\underset{\mathrm{IoU}=0.5}{\sum }}\frac{\mathrm{TP}@\mathrm{IoU}}{\mathrm{TP}@\mathrm{IoU}+\mathrm{FP}@\mathrm{IoU}}.$$

$$\mathrm{mAR}=\frac{1}{11}\overset{0.95}{\underset{\mathrm{IoU}=0.5}{\sum }}\frac{\mathrm{TP}@\mathrm{IoU}}{\mathrm{TP}@\mathrm{IoU}+\mathrm{FN}@\mathrm{IoU}}.$$

For tea picking machines, in addition to the localization performance of the model, the performance of real-time detection is also very important. A fast processing and decision speed is needed to improve picking efficiency. A common metric for evaluating speed is Frame Per Second (FPS). The higher the FPS, the more pictures can be processed per second, the faster the speed, and the more effective the model will be in operational situations.

The *F_β_* and FPS were chosen as the performance metrics for the shoot identification task. The relationship between the two and the model evaluation is that the larger the value, the better the model.

#### Evaluation of the position for tea shoots picking points

Precision and recall were used as performance metrics for the picking point location:


$$\mathrm{precision}=\frac{\mathrm{ED}}{\mathrm{ED}+\mathrm{ND}},$$

$$\mathrm{recall}=\frac{\mathrm{ED}}{\mathrm{ED}+\mathrm{EN}},$$

where ED, EN, and ND are calculated from the confusion matrix, the row sum of the confusion matrix represents the number of true markers of the picking point, and the column sum represents the number of predicted markers of the picking point. In this paper, P represents the positive examples, and N represents the negative examples. The specific representation is shown in Table 1.

**Table 1:** Definition of confusion matrix.

Confusion matrix		Prediction category (Picking point detected)	
		P	N
Actual category (Picking point exists)	P	ED (Picking point exists and detected)	EN (Picking point exists but not detected)
	N	ND (Pick point does not exist but is detected)	/

## Results and discussion

The experimental computing platform is a Tesla V100 graphics processing unit (GPU) with 16 GB of video memory (NVIDIA, Santa Clara, CA, United States). The experimental training tasks are pre-trained based on the COCO dataset, which is a large-scale dataset used for various tasks, such as image classification, object detection, and image segmentation (Lin et al., 2014). By adjusting the parameters, the optimal model was obtained with the best learning rate, batch size, and backbone. Meanwhile, the MR3P-TS model was compared with several mainstream target detection model including YOLOv3 (Redmon and Farhadi, 2018) and FasterRCNN (Ren et al., 2017), and showed the practical performance on the testing dataset.

### Parameter adjustment experiment

In order for the model to achieve the best results, it was necessary to design an experiment to tune the hyperparameters, such as the epoch number, the grid structure, and some function choices in the network. The sources of adjustment parameters can be divided into data processing, training, and network parameters. In this experiment, a set of parameters with the best performance was determined by testing different learning rates and batch sizes for the MR3P-TS model.

**Figure 5:**
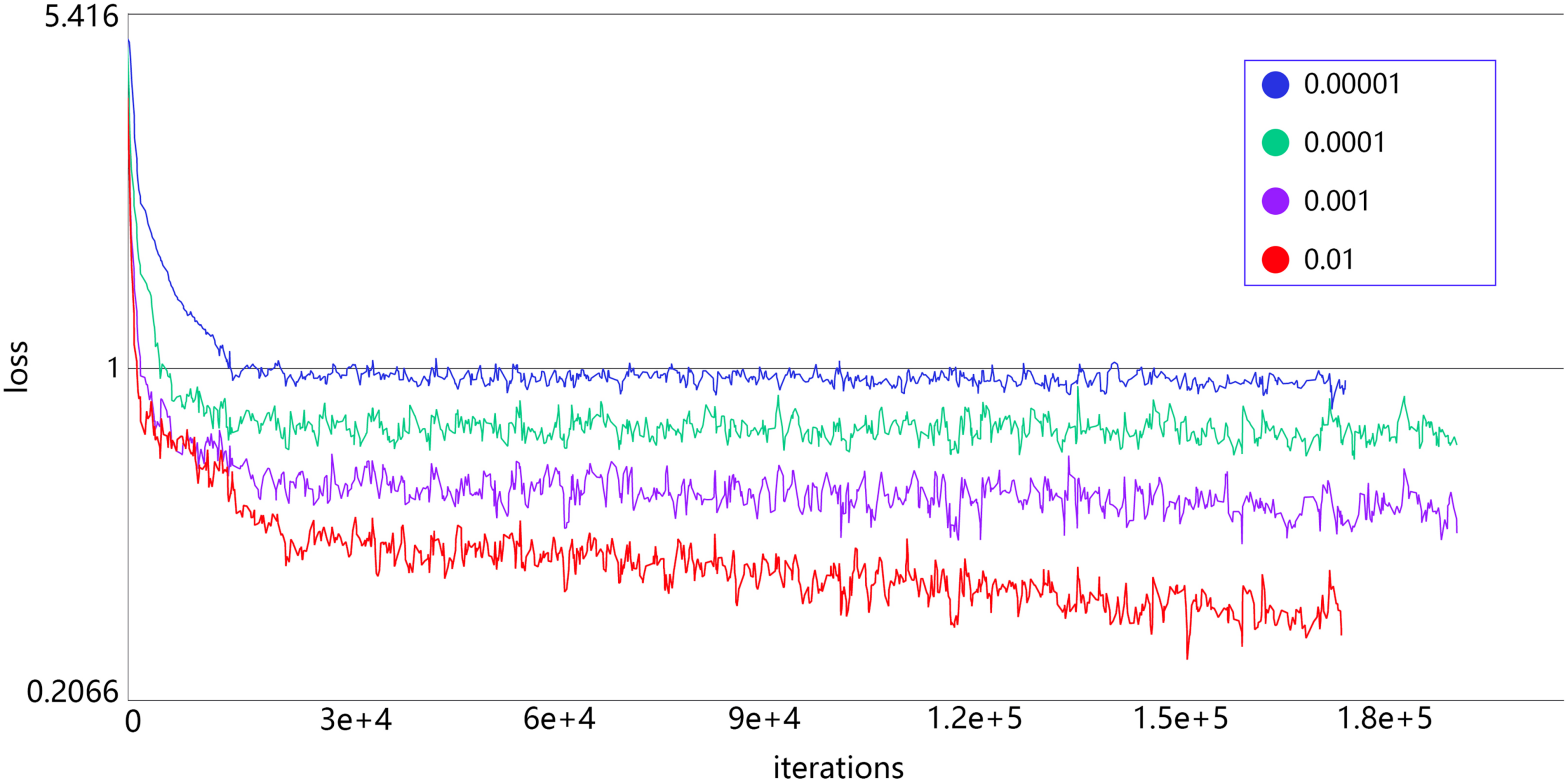
The decline of the loss curve of different learning rates during the training process.

The decrease of the loss curve of the models with different learning rates during the training process is shown in Figure 5. A smaller learning rate, although it is possible for the loss value to drop even lower, converges significantly slower than other models with larger learning rates, and is more likely to fall into the local minima. So, the learning rate in this group of test values was set to 0.01.

The evaluation results of different batch sizes in the dataset are shown in Table 2. The training time remained at the same level due to the parallel computing power of the GPU. As the batch size increased, the evaluation data of the model in the training set and the test set declined. This is because a large batch size needed more training data to update the gradient, which means that the larger updated step size was easier to converge to the sharp minima (Keskar et al., 2016). In the model training, the optimal batch size was 1 in this study.

**Table 2:** Evaluation results of different batch size.

Batch size	mAP	mAR	Training time
1	0.511	0.558	1 h 4 m (60 epoch)
2	0.389	0.441	1 h 35 m (100 epoch)
4	0.280	0.372	1 h 17 m (100 epoch)
8	0.274	0.374	1 h 21 m (100 epoch)
16	0.286	0.360	1 h 25 m (100 epoch)

**Figure 6:**
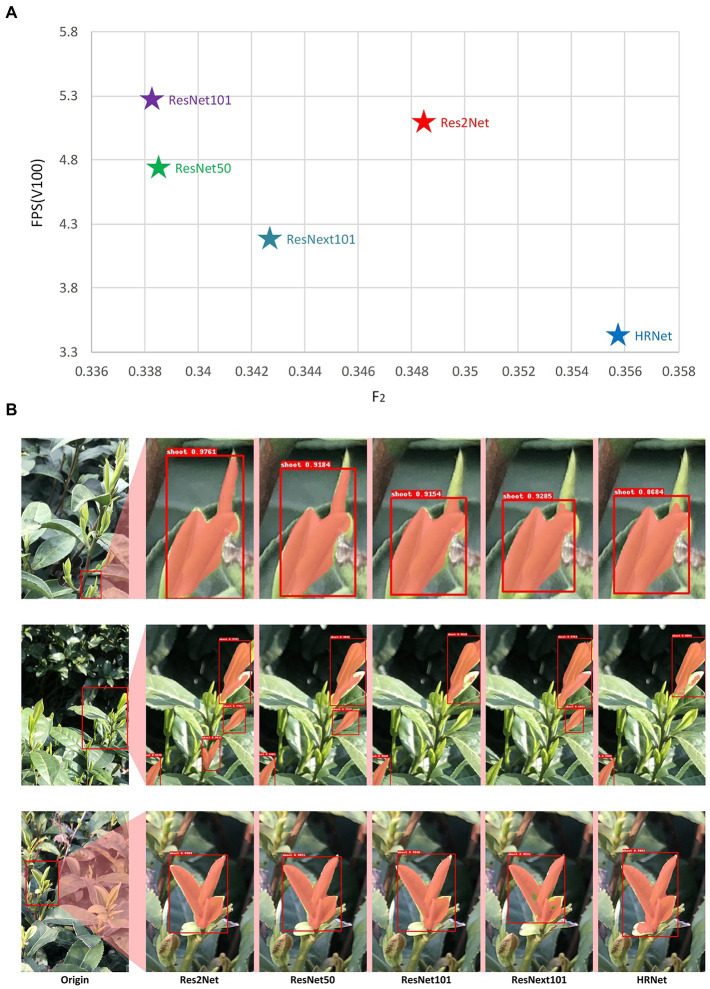
Evaluation results of different backbone **(A)** the relationship between F_2_ and FPS of model with different backbones, **(B)** the practical performance of models with different backbones on selected areas of a larger scene.

The evaluation results on the different backbone datasets are shown in Figure 6. The detection speed of the Res2Net network was slower than that of the ResNet101 network with similar F2 values, and the detection speed of HRNet was the slowest. From the detection results of the model, the HRNet was the best, but it cannot meet the requirements for real-time detection. Although the effect of Res2Net was slightly worse than HRNet, it was much higher than other networks. Therefore, combining the above two indicators, the optimal backbone in the experiment was Res2Net.

Figure 6B shows the actual detection results of different backbones for several example images. As shown in Figures 6B,C, there were holes (non-masked areas) within the identification results of the shoot mask when using ResNext101, which resulted in incomplete identification of the shoots. In the recognition results shown in Figure 6B, ResNet101 and HRNet had many missed detections. The results of ResNet50 were similar to Res2Net, but Res2Net’s FPS and F2 indicators were better than ResNet50. As a result of these tests, the learning rate was set to 0.01, batch size to 1, and Res2Net was selected as the backbone network for all subsequent image analyses with the MR3P-TS method.

### Comparison results of segmentation methods for tea shoots detection

The MR3P-TS model was compared with the YOLOv3 and Faster R-CNN algorithms for tea shoot location and segmentation to verify that the MR3P-TS model had better performance on the missed detection and overlapping problems.

In the actual picking operation, the method of detecting the same area from multiple angles can be adopted to solve the problem of tea shoot obscuration, so the focus here was on how well the segmentation approach detected shoots, rather than the reduction of missed shoots. Therefore, the F2 statistic was used as the primary performance measure as the accuracy was considered more important than the recall rate. From the data of the study results (Table 3), the MR3P-TS model proposed in this paper obtained an mAP of 0.449 and an F2 value of 0.313 in the test set. The F2 values of YOLOv3 and Faster R-CNN algorithms were 0.350 and 0.317, respectively.

**Table 3:** Recognition results of several models.

Model	Evaluation indicators		
	mAP	mAR	*F*_2_
MR3P-TS	0.449	0.544	0.313
YOLOv3	0.484	0.615	0.350
Faster R-CNN	0.446	0.555	0.317

**Figure 7:**
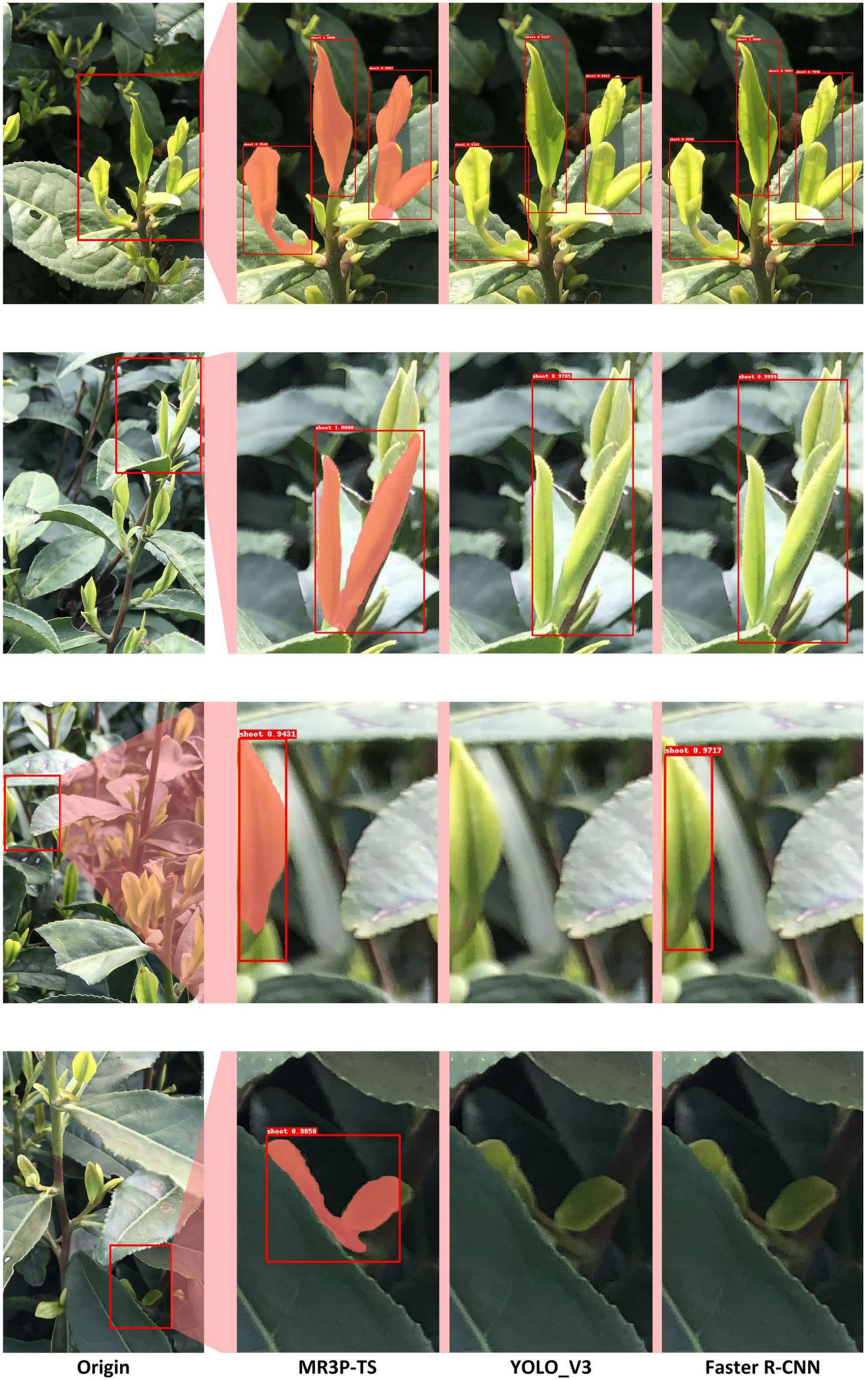
Example illustrating local details of the instance segmentation results of the MR3P-TS, YOLO_V3, and Faster R-CNN models.

The prediction results of the various models obtained from the testing set were visualized and examples are shown in Figure 7. The Faster R-CNN model had obvious target overlap, and the YOLOv3 model had two obvious missed detection phenomena. In contrast, the proposed MR3P-TS model actually performed better in the detection task of overlapping objects and edge objects. To sum up, although the MR3P-TS model still needs to be optimized in terms of data indicators, its performance was excellent in the actual tests. Therefore, it can be considered that the proposed MR3P-TS model was more suitable for solving the problem of tea shoot identification and picking point location compared to the well-known YOLOv3 and Faster R-CNN methods.

### Picking point position result

**Figure 8:**
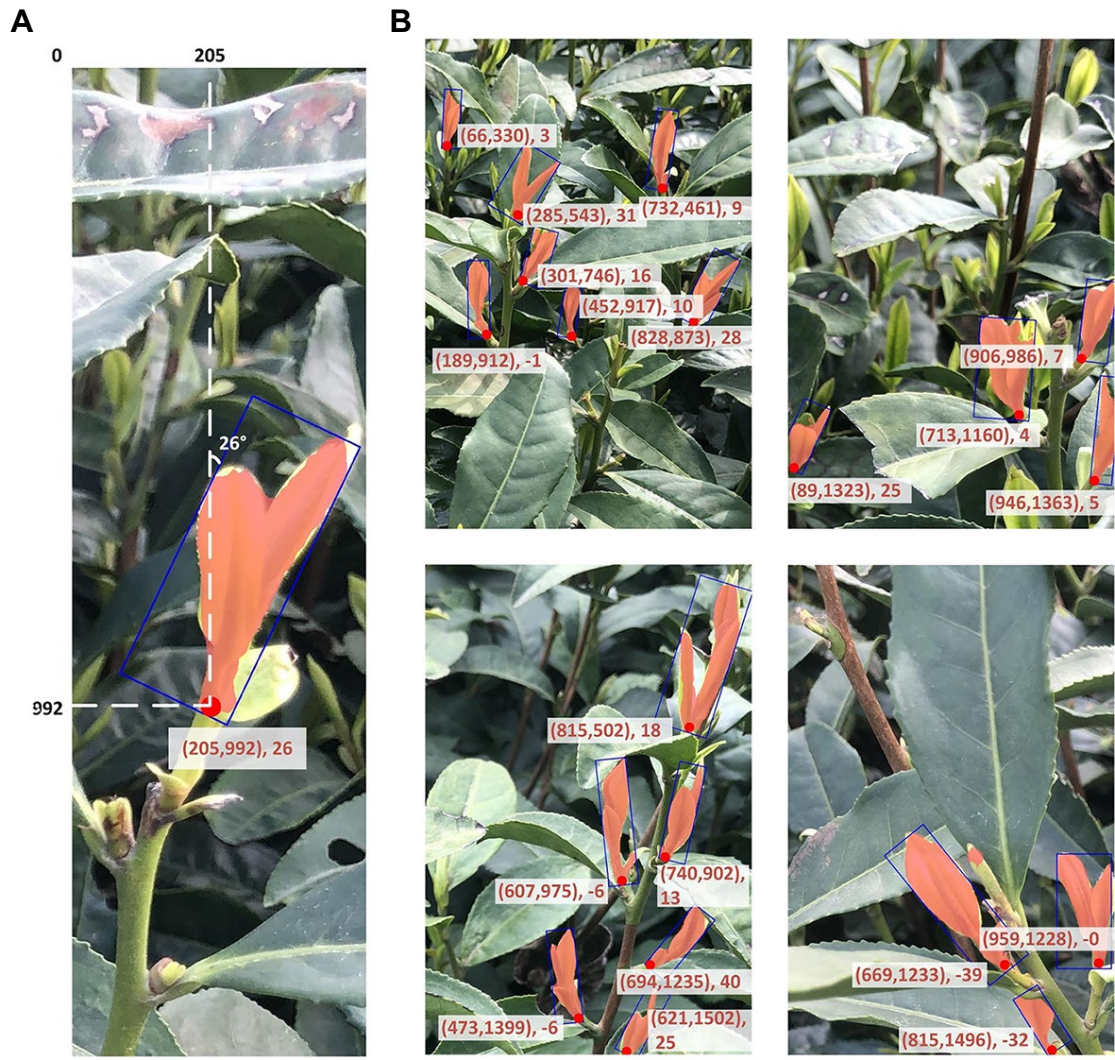
Examples of the resulting outcome of the MR3P-TS model **(A)** a single sample example of the shoot output, and **(B)** four images showing multiple tea shoots and picking point identification from the MR3P-TS model.

An example of the final result of the model evaluation with shoot identification and picking point positioning identified from the independent testing set is shown in Figure 8 (more detection results listed in the supplemental material). With the exception of some unfocused areas in the images, the model produced accurate representations of the shoot segmentation in complex scenes, and generated the coordinates of the two-dimensional picking point and the suggested knife angle. In total, the proposed MR3P-TS model identified 128 picking points in 42 images within the testing set, of which 111 were correctly identified, and the precision of the picking point positioning was 0.949, and the recall was 0.910.

As shown in Figure 8A, the set in parentheses represented the position coordinates of the picking point relative to the upper left corner of the image, and the following values are the angle information, which was the angle between the bottom edge of the rectangular box and the shoot axis.

### General discussion

Compared with the indoor measurement images under ideal and stable lighting conditions, the shoot images acquired in the tea garden environment with complex background were affected by light and wind, which affects the accuracy of tea shoots detection. To further improve the accuracy of shoot recognition, it is intended to add an attention module to the model in future research. The attention mechanism has been shown to improve the accuracy of the model (Nie et al., 2020; Wang et al., 2022). At the same time, in order to apply the model to the actual mechanized tea picking task, the corresponding mechanical structure and a complete visual recognition system can be designed for the tea picking machine in the future, and the model can be deployed to the development board to achieve fine tea picking.

In order to apply the model to the actual mechanized tea picking task, the model can be deployed to the development board, and the corresponding mechanical structure and complete visual recognition system can be designed for the tea picking machine to realize the fine picking of tender shoots. At present, some literature has been reported to use binocular depth cameras to collect field fruit images, and design a fruit spatial positioning system (Zhang et al., 2021). Some other researchers have proposed methods of connecting the manipulator with the tea picking machine, which provides theoretical support for this line of thinking (Yang et al., 2021). However, to realize a fully automated harvesting system there are still many aspects to work on, such as how to integrate the deployed model with the depth information provided by the binocular camera and how to debug the manipulator and other structures.

## Conclusion

In this study, a novel approach was proposed to identify the contour and counting of tender tea shoots and locate the picking points under field conditions. It is a three-step model using Res2Net as the backbone network to generate candidate regions, extract features, discriminate feature categories, and correct the position of candidate boxes, and finally extract masks for localization of picking points. By using an image dataset containing different lighting information for the model learning, the MR3P-TS model can directly use the two-dimensional mask obtained from the parallel prediction mask branch for the localization of the picking points. In the test set, the proposed MR3P-TS model achieved an mAP of 0.449 and an F2 value of 0.313 in shoot identification, and achieved a precision of 0.949 and a recall of 0.910 in localization of picking points. The samples of the multi-target overlap in the target detection were obviously less than other target detection algorithms with better numerical values and better actual engineering effect. The MR3P-TS algorithm has provided the necessary basic information for the realization of an automated tea picking machine. In future research, the intent is to design a reasonable tea picking scheme to better identify the shoots and locate the picking point and to encapsulate the process within a web interface and within an embedded imaging system. This will undoubtedly improve the automation level of tea production and contribute to the cause of agricultural science and technology.

## Data availability statement

The raw data supporting the conclusions of this article will be made available by the authors, without undue reservation.

## Author contributions

DC, LY, and JL conceptualized the topic of the article and designed this study. LY and JL collected the tea sprout data and marked the data. JL and XZ performed method validation and results collation. LY, JL, and DC did the original draft preparation. DC, JT, XX, JZ, and KW commented and edited the draft. All authors contributed to the article and approved the submitted version.

## Funding

This research was supported by the Zhejiang Provincial Natural Science Foundation of China under grant no. LGN19F030001, National Key R&D Program of China (2019YFE0125300); and Zhejiang Agricultural Cooperative and Extensive Project of Key Technology (2020XTTGCY04-02 and 2020XTTGCY01-05).

## Conflict of interest

The authors declare that the research was conducted in the absence of any commercial or financial relationships that could be construed as a potential conflict of interest.

## Publisher’s note

All claims expressed in this article are solely those of the authors and do not necessarily represent those of their affiliated organizations, or those of the publisher, the editors and the reviewers. Any product that may be evaluated in this article, or claim that may be made by its manufacturer, is not guaranteed or endorsed by the publisher.
