# Supplementary figures and images for: Identification and picking point positioning of tender tea shoots based on MR3P-TS model
Source: Front Plant Sci. 2022 Aug 12;13:962391. doi: 10.3389/fpls.2022.962391 (PMC9414667; doi:10.3389/fpls.2022.962391)

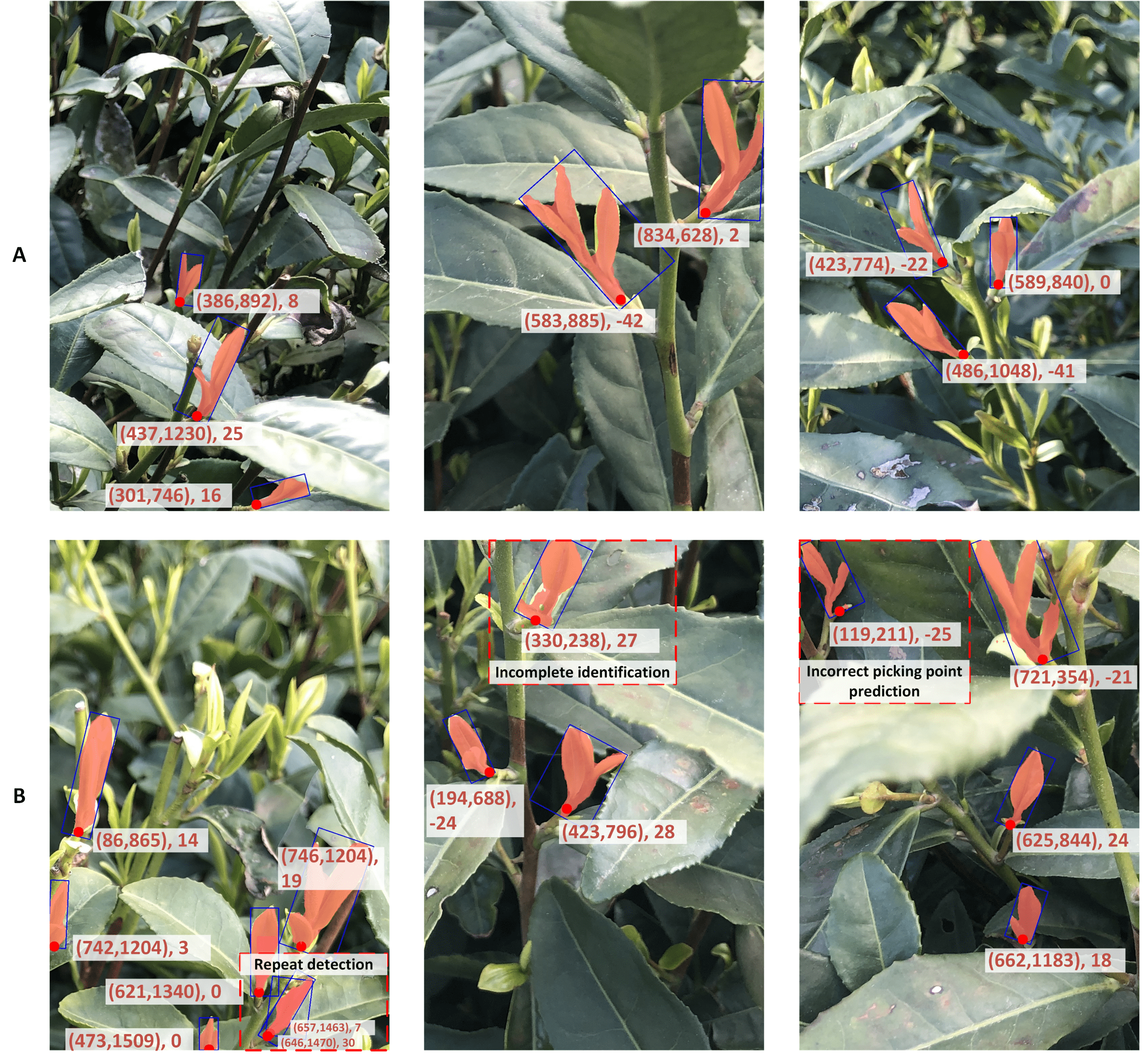

Supplement: Supplementary file 1 [file Image_1.png]
